# Assessing the performance of a suite of machine learning models for daily river water temperature prediction

**DOI:** 10.7717/peerj.7065

**Published:** 2019-06-04

**Authors:** Senlin Zhu, Emmanuel Karlo Nyarko, Marijana Hadzima-Nyarko, Salim Heddam, Shiqiang Wu

**Affiliations:** 1 State Key Laboratory of Hydrology-Water Resources and Hydraulic Engineering, Nanjing Hydraulic Research Institute, Nanjing, China; 2 Faculty of Electrical Engineering, Computer Science and Information Technology Osijek, University J.J. Strossmayer in Osijek, Osijek, Croatia; 3 Faculty of Civil Engineering Osijek, University J.J. Strossmayer in Osijek, Osijek, Croatia; 4 Faculty of Science, Agronomy Department, Hydraulics Division, Laboratory of Research in Biodiversity Interaction Ecosystem and Biotechnology, University 20 Août 1955, Skikda, Algeria

**Keywords:** River water temperature, Artificial neural network, Flow discharge, Decision tree, Air temperature, Gaussian process regression

## Abstract

In this study, different versions of feedforward neural network (FFNN), Gaussian process regression (GPR), and decision tree (DT) models were developed to estimate daily river water temperature using air temperature (*T_a_*), flow discharge (*Q*), and the day of year (*DOY*) as predictors. The proposed models were assessed using observed data from eight river stations, and modelling results were compared with the air2stream model. Model performances were evaluated using four indicators in this study: the coefficient of correlation (R), the Willmott index of agreement (d), the root mean squared error (RMSE), and the mean absolute error (MAE). Results indicated that the three machine learning models had similar performance when only *T_a_* was used as the predictor. When the day of year was included as model input, the performances of the three machine learning models dramatically improved. Including flow discharge instead of day of year, as an additional predictor, provided a lower gain in model accuracy, thereby showing the relatively minor role of flow discharge in river water temperature prediction. However, an increase in the relative importance of flow discharge was noticed for stations with high altitude catchments (Rhône, Dischmabach and Cedar) which are influenced by cold water releases from hydropower or snow melting, suggesting the dependence of the role of flow discharge on the hydrological characteristics of such rivers. The air2stream model outperformed the three machine learning models for most of the studied rivers except for the cases where including flow discharge as a predictor provided the highest benefits. The DT model outperformed the FFNN and GPR models in the calibration phase, however in the validation phase, its performance slightly decreased. In general, the FFNN model performed slightly better than GPR model. In summary, the overall modelling results showed that the three machine learning models performed well for river water temperature modelling.

## Introduction

Water temperature is one of the key indicators to determine the overall health of aquatic ecosystems since it impacts various physical and bio-chemical processes in rivers (Caissie, 2006). Clear examples are the significant impact of stream water temperature on dissolved oxygen dynamics (Marzadri, Tonina & Bellin, 2013) and algae growth (Goldman & Carpenter, 1974). Understanding the processes regulating water temperature and how thermal regimes have changed in the past as well as how they can be modified in the future is therefore of utmost importance for ecological applications.

This is particularly relevant considering that the increase of air temperature as a result of climate change, extreme events and anthropogenic pressures concur in impacting river thermal dynamics (Nelson & Palmer, 2007; Mantua et al., 2010; Cai et al., 2018; Piccolroaz et al., 2018). Ongoing warming of river water temperature has been observed by several authors in the past decades as a consequence of global warming (Moatar & Gailhard, 2006; Acuña et al., 2008; Tian & Yang, 2016; Weyhenmeyer et al., 2017), and according to the Report of European Environment Agency (2012), which stated that water temperature of some European rivers and lakes has increased from 1 to 3 °C during the last century, this trend is expected in the future as well. Besides the significant role of climatic change, anthropogenic activities (land use change, damming, thermal releases, etc.) can also strongly affect river thermal dynamics (Ozaki, Fukushima & Kojiri, 2008; Hester & Doyle, 2011; Casado et al., 2013; Wang et al., 2017; Kędra & Wiejaczka, 2018).

**Figure 1:**
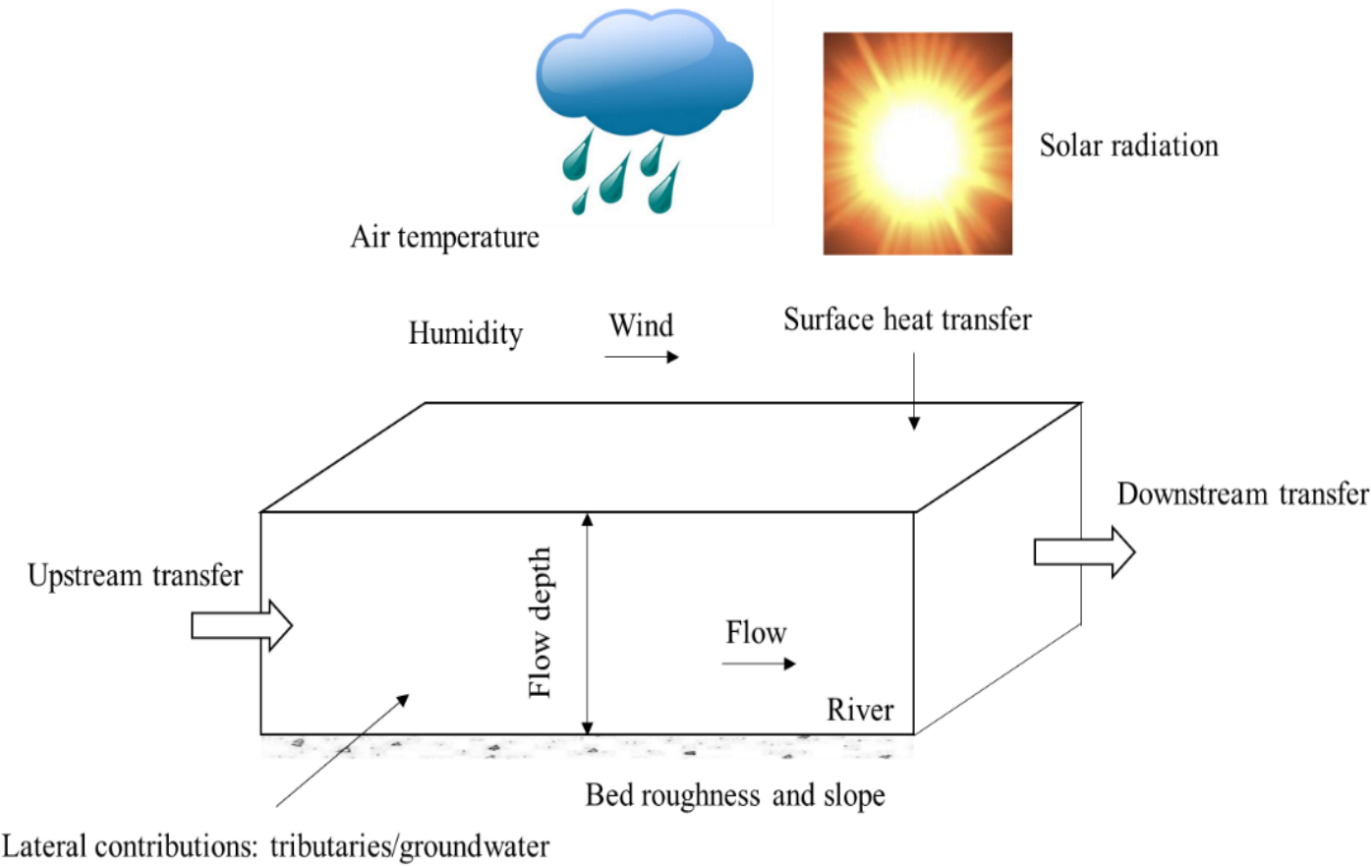
Schematic view of meteorological parameters and flow processes and their impacts on water temperatures in a river.

Thermal dynamics in rivers depend on multiple factors (Blaen et al., 2013; Cai et al., 2018). River temperature follows a diurnal cycle and a seasonal cycle, which are the result of heat inputs and outputs under specific meteorological (air temperature, humidity, wind and solar radiation) and hydrological conditions (depth, flow discharge and volume of groundwater exchange), as schematized in Fig. 1. Despite that many factors affect river water temperatures, studies have demonstrated that there exists a strong relationship between water temperature and air temperature (Webb, Clack & Walling, 2003; Hadzima-Nyarko, Rabi & Šperac, 2014; Toffolon & Piccolroaz, 2015). However, the modelling of their relationship is not as trivial as it seems, especially when climatic change is to be considered (Piotrowski et al., 2016).

The modeling and forecasting of water temperature under different spatial and temporal scales is usually solved by two types of models: deterministic and statistical models. Deterministic models apply energy budget approaches to predict river water temperature (Sinokrot & Stefan, 1993; Zhu, Du & Luo, 2018; Du et al., 2018), while statistical models mainly predict river water temperature using air temperature data (Stefan & Preud’homme, 1993; Mohseni & Stefan, 1999; Webb, Clack & Walling, 2003; Caissie, 2006). Another group of statistical models are the so called non-parametric models. Artificial Neural Networks (ANN), which belong to this group, have demonstrated to be a good mathematical tool to characterize, model and predict a great amount of non-linear processes, and their applications in river water temperature predictions have been well documented (Sahoo, Schladow & Reuter, 2009; Hadzima-Nyarko, Rabi & Šperac, 2014; Deweber & Wagner, 2014; Rabi, Hadzima-Nyarko & Sperac, 2015; Temizyurek & Dadasercelik, 2018; Zhu, Nyarko & Hadzima-Nyarko, 2018; Zhu et al., 2019). The Gaussian process regression (GPR) model has been widely used in engineering (Sun, Wang & Xu, 2014; Kang et al., 2015; Tan et al., 2016). Its applications in hydrology are also quite impressive (Rasouli, Hsieh & Cannon, 2012; Grbić, Kurtagić & Slišković, 2013; Sun, Wang & Xu, 2014; Zhu, Nyarko & Hadzima-Nyarko, 2018). Currently, GPR models have only been applied for river temperature prediction by Grbić, Kurtagić & Slišković (2013) and Zhu, Nyarko & Hadzima-Nyarko (2018), and modelling results showed that GPR models can be efficiently used for river water temperature predictions. Decision Tree (DT) models are supervised machine learning approaches, which are very popular in machine learning (Pradhan, 2013; Rutkowski et al., 2014; Tsangaratos & Ilia, 2016). They have also been frequently used in hydrology (Balk & Elder, 2000; Schärer, Page & Beven, 2006; Tehrany, Pradhan & Jebur, 2013; Kumar et al., 2013), and have been applied only once in water temperature modeling (Zhu, Nyarko & Hadzima-Nyarko, 2018). Further investigations about these robust approaches in river water temperature modelling are needed since accurate simulation of river water temperature plays an important role in water resources management. Previously, ANN, GPR and DT models were compared in water temperature modeling of the Missouri River, USA (Zhu, Nyarko & Hadzima-Nyarko, 2018) using only air temperature (T_a_) as a predictor. However, some researches have also tried to model river water temperature by considering multiple factors, such as river flow discharge (Webb, Clack & Walling, 2003; Arismendi et al., 2014; Laanaya, St-Hilaire & Gloaguen, 2017), solar radiation (Sahoo, Schladow & Reuter, 2009), riparian shade (Johnson, Wilby & Toone, 2014), landform attributes, and forested land cover (Deweber & Wagner, 2014). Air temperature and river flow discharge are generally the most available variables for modeling temperatures in rivers, and they have been shown to have the greatest impact on water temperature (Webb, Clack & Walling, 2003). In this research, ANN, GPR and DT models were developed for eight river stations characterized by different hydrological conditions using *T*_*a*_, flow discharge (*Q*) and day of the year (*DOY*) as predictors. Modelling performances were assessed by comparing with air2stream, a hybrid statistical-physical based model (Toffolon & Piccolroaz, 2015; Piccolroaz et al., 2016). The aim of this study is to contribute to water temperature modelling for river systems.

## Materials & Methods

### Study sites and data

The measurements of daily water temperature (*T*_*w*_), flow discharge (*Q*) and air temperature (*T*_*a*_) were obtained from seven rivers in Europe and USA: (i) one river in Croatia, (ii) three rivers in Switzerland, and (iii) three rivers in USA. The main characteristics of the rivers with the period of records, and the calibration and validation periods are briefly summarized in Table 1.

**Table 1:** Characteristics of river and meteorological stations.

River Name	River station name/number	Catchment area (km^2^)	Calibration period	Validation period	Meteorological station name	Distance from river station (km)
Drava	Botovo	31038	1991–2008	2009–2016	Koprivnica	11.8
Drava	Donji Miholjac	37142	1993–2008	2009–2016	Donji Miholjac	1
Mentue	Yvonand	105	2002–2009	2010–2012	Mathod	12.7
Rhône	Sion	3373	1984–2003	2004–2013	Sion	2.14
Dischmabach	Davos	43.3	2004–2009	2010–2012	Davos	4.9
Cedar	USGS12119000	477	2001–2012	2013–2017	USW00024233	9.4
Fanno	USGS14206950	82	2003–2012	2013–2017	USW00024229	24.1
Irondequoit	USGS423205010	368	2005–2013	2014–2017	USW00014768	13.7

### The rescaled adjusted partial sums (RAPS) method

In order to detect and quantify trends and fluctuations in time series, the widely used rescaled adjusted partial sums (RAPS) method (Bonacci, Trninić & Roje-Bonacci, 2008; Bonacci & Oskoruš, 2010; Basarin et al., 2016) was employed in this study. The RAPS method can highlight trends, shifts, data clustering, irregular fluctuations and periodicities in the time series (Bonacci, Trninić & Roje-Bonacci, 2008), and is defined as:

(1) \begin{eqnarray*}RAP{S}_{i}=\sum _{n=1}^{i} \frac{{T}_{n}-{T}_{m}}{{S}_{T}} \end{eqnarray*}

Descriptions of the variables are summarized in Table 2.

**Table 2:** Summary of variables.

Variable	Description
*T*_*m*_	The mean value of the time series
*S*_*T*_	The standard deviation
*T*_*n*_	The value of a sample, *n* = 1, 2…, *N*
*N*	The number of values in the time series
*T*_*w*_	Water temperature (°C)
*T*_*a*_	Air temperature (°C)
*Q*	Flow discharge (m^3^/s)
*DOY*	Day of the year (1 to 365 for a regular year or 1 to 366 for a leap year)
*θ*	The dimensionless flow discharge
*t*	Time
*t*_*y*_	The duration of a year
*a*_1_-*a*_8_	Parameters used in the Air2stream model, which are estimated through model calibration
*O*_*i*_	The observed water temperatures
*P*_*i*_	The predicted water temperatures
*O*_*m*_	The average values of *O*_*i*_
*P*_*m*_	The average values of *P*_*i*_

### Machine learning models

In this study, we compared three machine learning models: feedforward artificial neural network (FFNN), GPR and DT. Detailed descriptions of these models can be found in Zhu, Nyarko & Hadzima-Nyarko (2018).

### Air2stream

Air2stream is a hybrid model which combines a physically based structure with a stochastic calibration of parameters (Toffolon & Piccolroaz, 2015; source code available at https://github.com/spiccolroaz/air2stream). The model has been tested in various river systems, and generally it performs well. The equation for the air2stream model can be expressed as (the 8-parameter version):

(2) \begin{eqnarray*} \frac{d{T}_{w}}{dt} = \frac{1}{{\theta }^{{a}_{4}}} [{a}_{1}+{a}_{2}{T}_{a}-{a}_{3}{T}_{w}+\theta ({a}_{5}+{a}_{6}\cos \nolimits (2\pi ( \frac{t}{{t}_{y}} -{a}_{7}))-{a}_{8}{T}_{w})]\end{eqnarray*}

Descriptions of the variables can be found in Table 2. In this model version, *T*_*w*_ is estimated from *T*_*a*_, *Q* and a sinusoidal term.

Assuming that the effect of flow discharge can be approximately retained using only a constant value and by combining the constant terms and those proportional to *T*_*w*_, a 5-parameter model version can be obtained (Toffolon & Piccolroaz, 2015). In this model version, *T*_*w*_ is estimated from *T*_*a*_ and a sinusoidal term.

(3) \begin{eqnarray*} \frac{d{T}_{w}}{dt} ={a}_{1}+{a}_{2}{T}_{a}-{a}_{3}{T}_{w}+{a}_{6}\cos \nolimits (2\pi ( \frac{t}{{t}_{y}} -{a}_{7}))\end{eqnarray*}

Disregarding the second term on the right hand of Eq. (2) and assuming that the effect of the flow discharge can be approximately retained using only a constant value, a 3-parameter model version can be obtained (Toffolon & Piccolroaz, 2015). In this model version, *T*_*w*_ is estimated from *T*_*a*_ only.

(4) \begin{eqnarray*} \frac{d{T}_{w}}{dt} ={a}_{1}+{a}_{2}{T}_{a}-{a}_{3}{T}_{w}\end{eqnarray*}

The detailed information about the model can be found in Toffolon & Piccolroaz (2015) and Piccolroaz et al. (2016).

### Performance indices

Model performances were evaluated using four indicators in this study: the coefficient of correlation (R), the Willmott index of agreement (d), the root mean squared error (RMSE), and the mean absolute error (MAE).

(5) \begin{eqnarray*}R= \left[ \frac{ \frac{1}{N} \sum _{i=1}^{N} \left( {\mathrm{O}}_{i}-{\mathrm{O}}_{m} \right) \left( {\mathrm{P}}_{i}-{\mathrm{P}}_{m} \right) }{\sqrt{ \frac{1}{N} \sum _{i=1}^{N}{ \left( {\mathrm{O}}_{i}-{\mathrm{O}}_{m} \right) }^{2}}\sqrt{ \frac{1}{N} \sum _{i=1}^{N}{ \left( {\mathrm{P}}_{i}-{\mathrm{P}}_{m} \right) }^{2}}} \right] \end{eqnarray*}

(6) \begin{eqnarray*}d=1- \frac{\sum _{i=1}^{N}{ \left( {\mathrm{P}}_{i}-{\mathrm{O}}_{i} \right) }^{\,2}}{\sum _{i=1}^{N}{ \left( \left\vert {\mathrm{P}}_{i}-{\mathrm{O}}_{m} \right\vert + \left\vert {\mathrm{O}}_{i}-{\mathrm{O}}_{m} \right\vert \right) }^{\,2}} \end{eqnarray*}

(7) \begin{eqnarray*}RMSE=\sqrt{ \frac{1}{N} \sum _{i=1}^{N}{ \left( {\mathrm{O}}_{i}-{\mathrm{P}}_{i} \right) }^{2}}\end{eqnarray*}

(8) \begin{eqnarray*}MAE= \frac{1}{N} \sum _{i=1}^{N} \left\vert {\mathrm{O}}_{i}-{\mathrm{P}}_{i} \right\vert \end{eqnarray*}

Descriptions of the variables can be found in Table 2.

### Model versions

The models were compared using three different versions: (i) version 1: FFNN1, GPR1 and DT1 using only *T*_*a*_ as input variable, (ii) version 2: FFNN2, GPR2 and DT2 with *T*_*a*_ and *DOY* as input variables, and (iii) version 3: FFNN3, GPR3 and DT3 with *T*_*a*_, *Q*, and *DOY* as input variables. For comparison, the 3-parameter, 5-parameter, and 8-parameter version air2stream models were used correspondingly.

## Results

### Seasonal dynamics of *T_a_*, *T_w_* and *Q*

Detailed statistics for the variables selected (*T*_*w*_, *Q* and *T*_*a*_) can be found in Table 3. Mean annual *T*_*w*_ for Rhône at Sion and Dischmabach at Davos are smaller compared with *T*_*w*_ for other river stations since Sion lies at the bottom of a populated Alpine valley, and Davos is located in a steep glacial valley (Dischma). Mean annual *T*_*a*_ for Dischmabach at Davos is extremely colder than the other stations. Mentue at Yvonand, Dischmabach at Davos, Fanno and Irondequoit have smaller annual mean flow discharge (*Q* < 10.0 m^3^/s).

**Table 3:** Basic statistical characteristics of the mean annual water temperature (*T*_w_ : °C), air temperature (*T*_a_: °C), and flow discharge (Q: m^3^/s) at the eight gauging and meteorological stations.

River station	Item	Average	Min	Max	Range
Botovo	*T*_*w*_	11.431	10.468	12.293	1.824
	*T*_*a*_	11.391	9.692	12.499	2.808
	*Q*	478.96	326.52	739.26	412.74
Donji Miholjac	*T*_*w*_	11.958	11.011	13.132	2.122
	*T*_*a*_	11.782	10.486	12.698	2.212
	*Q*	515.04	355.23	778.44	423.21
Yvonand	*T*_*w*_	9.718	9.063	10.253	1.190
	*T*_*a*_	9.919	9.046	10.349	1.302
	*Q*	1.397	0.652	2.015	1.363
Sion	*T*_*w*_	7.004	6.563	7.653	1.090
	*T*_*a*_	10.290	8.755	11.379	2.624
	*Q*	103.48	78.69	126.86	48.17
Davos	*T*_*w*_	4.296	4.041	4.564	0.523
	*T*_*a*_	3.786	2.743	4.763	2.020
	*Q*	1.683	1.457	1.979	0.522
Cedar	*T*_*w*_	10.453	9.541	11.814	2.273
	*T*_*a*_	11.593	10.587	12.866	2.279
	*Q*	20.237	13.043	27.450	14.407
Fanno	*T*_*w*_	12.773	11.954	13.925	1.970
	*T*_*a*_	12.602	11.671	13.708	2.037
	*Q*	1.349	0.701	1.896	1.195
Irondequoit	*T*_*w*_	10.851	9.886	11.784	1.897
	*T*_*a*_	9.807	8.425	11.060	2.634
	*Q*	4.218	2.986	5.841	2.855

**Figure 2:**
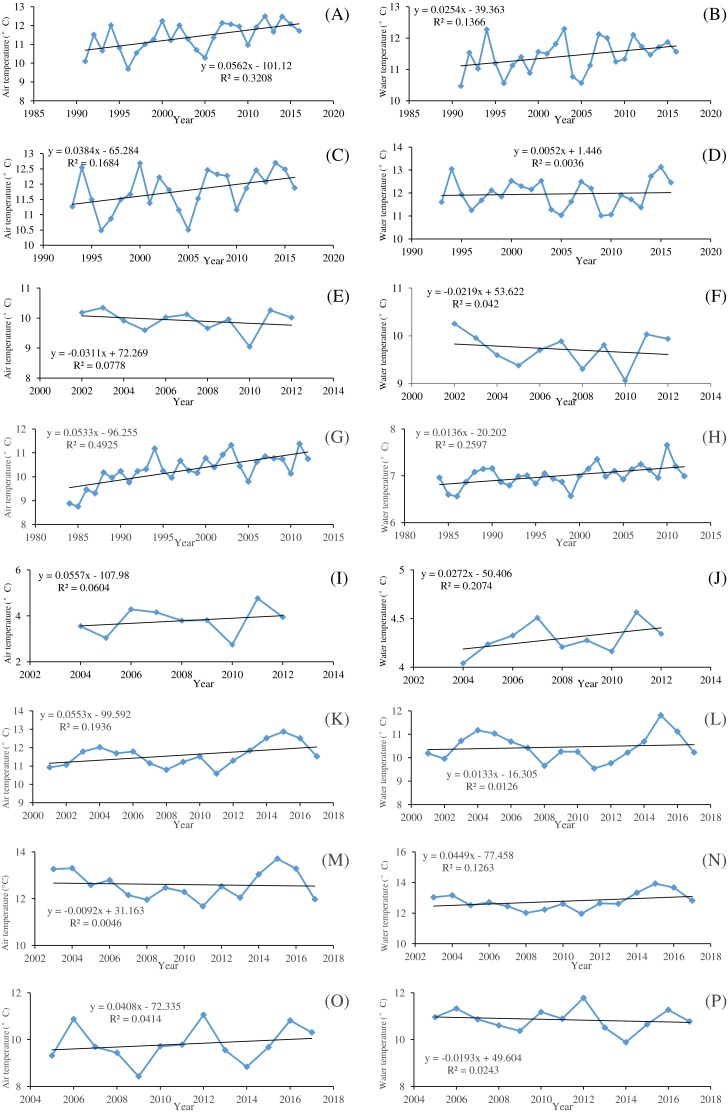
Temporal variations of the mean annual air temperature and water temperature of the eight river stations. (A) Drava at Botovo air temperature. (B) Drava at Botovo water temperature. (C) Drava at Donji Miholjac air temperature. (D) Drava at Donji Miholjac water temperature. (E) Mentue at Yvonand air temperature. (F) Mentue at Yvonand water temperature. (G) Rhône at Sion air temperature. (H) Rhône at Sion water temperature. (I) Dischmabach at Davos air temperature. (J) Dischmabach at Davos water temperature. (K) Cedar air temperature. (L) Cedar water temperature. (M) Fanno air temperature. (N) Fanno water temperature. (O) Irondequoit air temperature. (P) Irondequoit water temperature.

**Figure 3:**
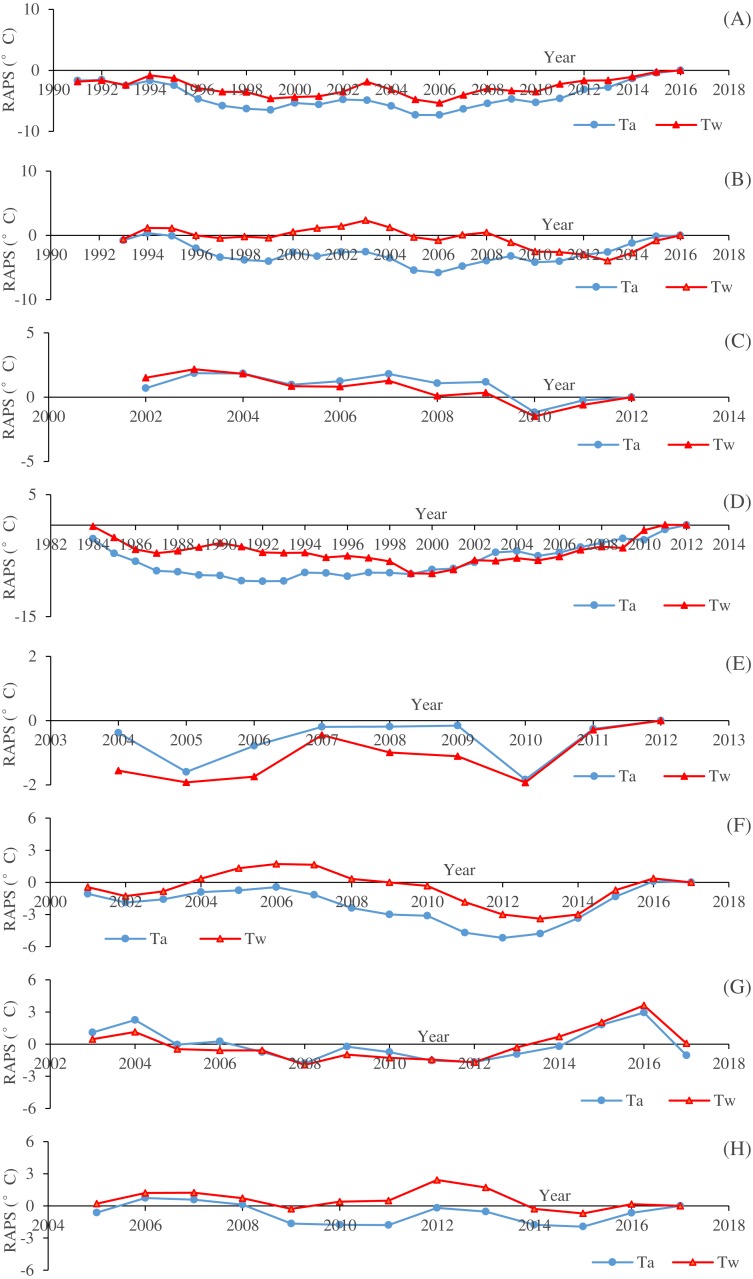
Time data series of the rescaled adjusted partial sums (RAPS) for the mean annual air temperature (Ta) and water temperature (Tw) of the eight river stations. (A) Drava at Botovo. (B) Drava at Donji Miholjac. (C) Mentue at Yvonand. (D) Rhône at Sion. (E) Dischmabach at Davos. (F) Cedar. (G) Fanno. (H) Irondequoit.

Figure 2 shows the temporal variations of annual averaged *T*_*w*_ and *T*_*a*_ for the studied river stations. It can be seen that the increasing rates of *T*_*w*_ are smaller than that of *T*_*a*_ in general. *T*_*a*_ displays an increasing trend except for Mentue at Yvonand and Fanno, and the increasing rates range from 0.0384 to 0.0562 °C/year. *T*_*w*_ displays an increasing trend except for Mentue at Yvonand and Irondequoit, and the increasing rates vary between 0.0052 and 0.0449 °C/year. Generally, *T*_*w*_ and *T*_*a*_ display the same varying pattern. However, for Fanno, *T*_*w*_ has an increasing trend with a decreasing trend of *T*_*a*_, while for Irondequoit, *T*_*w*_ has a decreasing trend with an increasing trend of *T*_*a*_, which indicates that the temporal variations of *T*_*w*_ may not be explained only by *T*_*a*_. It is shown in Fig. 3 that the studied river stations present different characteristics for RAPS values. For Drava at Botovo, increases of *T*_*w*_ and *T*_*a*_ started in 2006, and during the period 1991–2005, a trend of decreasing temperature is indicated (Fig. 3A). For Drava at Donji Miholjac, increases of *T*_*a*_ also started in 2006, however, a trend of decreasing *T*_*w*_ is evident until 2013, which explains the smaller increasing rate (0.0052 °C/year) at this station (Fig. 3B) compared with that of Botovo (0.0254 °C/year). A trend of decreasing *T*_*w*_ and *T*_*a*_ is shown for Mentue at Yvonand (Fig. 3C), confirming the results in Fig. 3C. For Rhône at Sion, increases of *T*_*w*_ and *T*_*a*_ started in 2000 and 1992 respectively, and during the periods 1984–1999 and 1984–1991, a trend of decreasing temperature is noticed (Fig. 3D). A trend of increasing *T*_*w*_ and *T*_*a*_ is shown for Dischmabach at Davos (Fig. 3E). For Cedar, increases of *T*_*w*_ and *T*_*a*_ started in started in 2013 and 2012 respectively, and during the periods 2001–2012 and 2001–2011, a trend of decreasing temperature is presented (Fig. 3F). For Fanno and Irondequoit, the trends of RAPS values are not quite significant (Figs. 3G and 3H).

**Figure 4:**
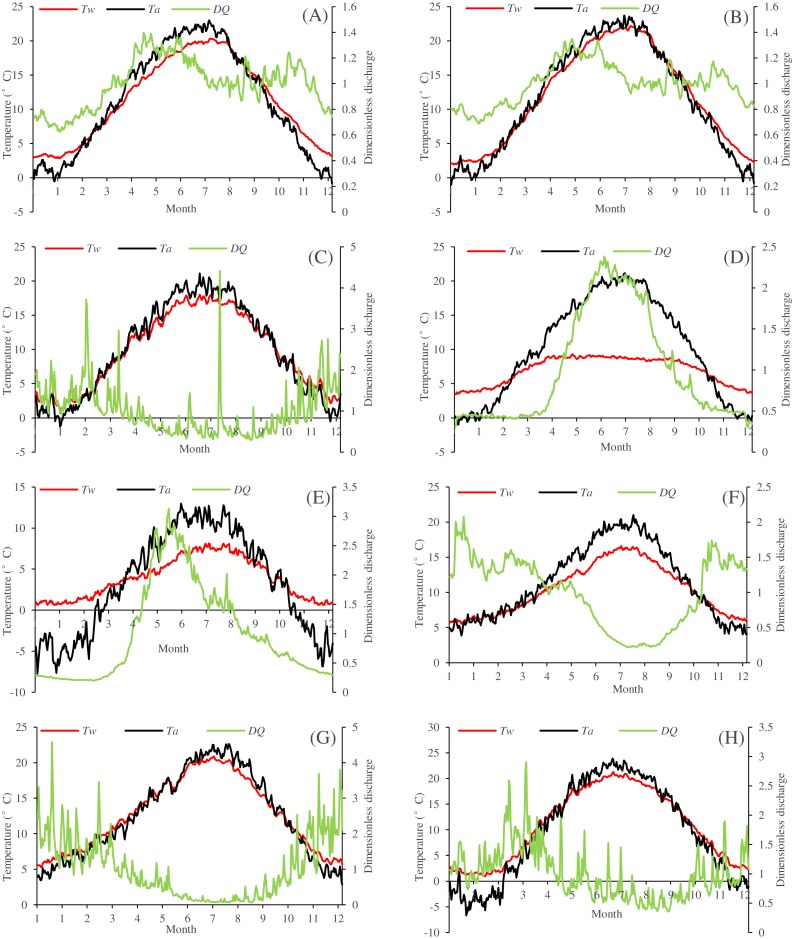
Climatological years for the eight river stations. (A) Drava at Botovo. (B) Drava at Donji Miholjac. (C) Mentue at Yvonand. (D) Rhône at Sion. (E) Dischmabach at Davos. (F) Cedar. (G) Fanno. (H) Irondequoit.

Figure 4 presents the seasonal dynamics of *T*_*w*_, *T*_*a*_ and dimensionless flow discharge (*DQ*: the ratio of daily flow discharge to annually averaged flow discharge) for the eight river stations through the climatological year. Results showed that the seasonal variations of *T*_*w*_ and *T*_*a*_ are almost synchronous for Drava at Botovo and Donji Miholjac, Mentue at Yvonand, Fanno and Irondequoit, indicating the insignificant role of *Q*. The damped responses of *T*_*w*_ to variations in *T*_*a*_ can be found in Rhône at Sion, Dischmabach at Davos and Cedar, especially for Rhône River at Sion since it is impacted by cold water releases from high altitude hydropower reservoir, indicating the significant role of *Q* in these stations.

### Model performances

All the four models (GPR, DT, FFNN and air2strteam) were applied and compared for the eight stations, and modelling performances were evaluated using RMSE, MAE, R and d values. The developed models were able to successfully predict *T*_*w*_ using *T*_*a*_, *Q*, and *DOY* as inputs. Tables 4–11 present the statistical indicators for the eight stations. For the eight studied stations, the FFNN, GPR and DT models performed with relatively similar accuracy when only *T*_*a*_ was used as predictor, with DT model performing slightly worse than FFNN and GPR. When *DOY* was included as model input, modelling performances of the three FFNN, GPR and DT models dramatically improved in terms of higher R, d and lower RMSE and MAE values (Tables 4–11). The comparison between the improvement in river *T*_*w*_ prediction obtained using model version 3 and version 2 indicates that the inclusion of discharge provided a lower gain than including *DOY* for all the cases (Tables 4–11). The relative importance of *Q* increases for the studied station in the Rhône, Dischmabach and Cedar Rivers, which suggests that the role of *Q* on river *T*_*w*_ modelling is more relevant for high altitude rivers (cold water release from hydropower reservoirs, snow melting and etc.). However, for lowland hydrological stations impacted by hydropower reservoirs, such as the Drava River at Botovo, the influence of *Q* is not that remarkable.

**Figure 5:**
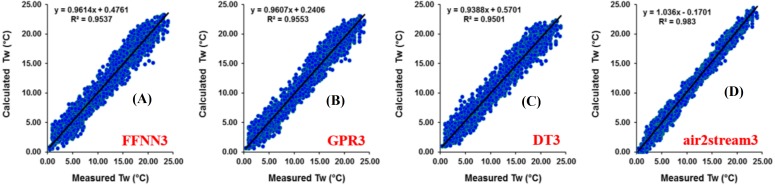
Scatterplot of measured versus calculated water *T_w_* at the Botovo River during the validation phase. (A) FFNN3. (B) GPR3. (C) DT3. (D) air2stream3.

Results at Botovo station are reported in Table 4. Figure 5 illustrates the scatterplot of measured and calculated values of *T*_*w*_ using the best models with *T*_*a*_, *Q*, and *DOY* as input variables (version 3). During the validation phase, inspection of statistical metrics for different models shows that the RMSE and MAE are lowest for the three versions of the air2strteam model (Table 4), and the best accuracy was obtained using air2strteam3 (RMSE = 0.891 °C and MAE = 0.724 °C). Averaged RMSE and MAE values for the three air2strteam models are 0.986 °C and 0.797 °C respectively. Compared to the three machine learning models, air2strteam3 is more accurate, followed by GPR3 (RMSE = 1.302 °C and MAE = 1.042 °C) and FFNN3 (RMSE = 1.307 °C and MAE = 1.040 °C) with similar accuracy, and finally the DT3 is ranked in the third place with the highest RMSE (1.366 °C) and MAE (1.086 °C) values respectively.

**Table 4:** Performances of different model versions in modelling water temperature (*T*_w_) for Botovo station.

Model version	Training (Calibration)				Validation			
	R	d	RMSE (°C)	MAE (°C)	R	d	RMSE (°C)	MAE (°C)
FFNN3	0.981	0.990	1.227	0.956	0.977	0.988	1.307	1.040
FFNN2	0.977	0.988	1.345	1.063	0.975	0.987	1.353	1.084
FFNN1	0.926	0.961	2.366	1.838	0.922	0.958	2.350	1.836
GPR3	0.980	0.990	1.236	0.970	0.977	0.988	1.302	1.042
GPR2	0.977	0.988	1.350	1.065	0.975	0.987	1.380	1.104
GPR1	0.926	0.960	2.370	1.841	0.922	0.959	2.366	1.844
DT3	0.989	0.994	0.956	0.729	0.975	0.987	1.366	1.086
DT2	0.984	0.992	1.112	0.859	0.972	0.986	1.445	1.148
DT1	0.929	0.962	2.320	1.802	0.919	0.957	2.424	1.899
air2stream3	0.990	0.995	0.876	0.680	0.991	0.995	0.891	0.724
air2stream2	0.987	0.994	1.002	0.805	0.988	0.993	1.000	0.804
air2stream1	0.986	0.993	1.046	0.838	0.987	0.993	1.066	0.863

**Table 5:** Performances of different model versions in modelling water temperature (T_w_) for Donji Miholjac station.

Model version	Training (Calibration)				Validation			
	R	d	RMSE (°C)	MAE (°C)	R	d	RMSE (°C)	MAE (°C)
FFNN3	0.984	0.992	1.290	1.014	0.971	0.986	1.690	1.337
FFNN2	0.979	0.989	1.471	1.172	0.972	0.986	1.677	1.336
FFNN1	0.934	0.965	2.579	2.012	0.928	0.962	2.645	2.063
GPR3	0.982	0.991	1.344	1.053	0.975	0.987	1.592	1.248
GPR2	0.979	0.989	1.481	1.178	0.972	0.985	1.694	1.344
GPR1	0.933	0.965	2.584	2.015	0.928	0.961	2.671	2.090
DT3	0.990	0.995	1.014	0.778	0.972	0.985	1.668	1.314
DT2	0.986	0.993	1.190	0.932	0.969	0.984	1.783	1.422
DT1	0.937	0.967	2.510	1.947	0.925	0.960	2.732	2.134
air2stream3	0.993	0.996	0.876	0.688	0.987	0.992	1.247	0.969
air2stream2	0.991	0.996	0.959	0.771	0.985	0.991	1.310	1.015
air2stream1	0.989	0.995	1.057	0.843	0.984	0.991	1.370	1.068

**Table 6:** Performances of different model versions in modelling water temperature (T_w_) for Mentue station.

Model version	Training (Calibration)				Validation			
	R	d	RMSE (°C)	MAE (°C)	R	d	RMSE (°C)	MAE (°C)
FFNN3	0.991	0.995	0.790	0.610	0.988	0.994	0.913	0.685
FFNN2	0.988	0.994	0.901	0.708	0.986	0.993	0.984	0.738
FFNN1	0.978	0.989	1.210	0.927	0.975	0.987	1.307	0.982	
GPR3	0.990	0.995	0.808	0.620	0.988	0.994	0.900	0.670	
GPR2	0.987	0.994	0.913	0.718	0.986	0.993	0.990	0.750	
GPR1	0.977	0.989	1.216	0.930	0.975	0.987	1.311	0.992	
DT3	0.994	0.997	0.611	0.463	0.987	0.993	0.947	0.728	
DT2	0.993	0.996	0.694	0.539	0.985	0.992	1.034	0.790	
DT1	0.985	0.992	0.993	0.758	0.973	0.986	1.364	1.038	
air2stream3	0.994	0.997	0.634	0.487	0.992	0.995	0.780	0.553	
air2stream2	0.993	0.997	0.666	0.517	0.991	0.995	0.800	0.573	
air2stream1	0.991	0.995	0.793	0.610	0.988	0.993	0.933	0.679	

**Table 7:** Performances of different model versions in modelling water temperature (T_w_) for Rhne station.

Model version	Training (Calibration)				Validation			
	R	d	RMSE (°C)	MAE (°C)	R	d	RMSE (°C)	MAE (°C)	
FFNN3	0.966	0.982	0.547	0.422	0.944	0.971	0.763	0.530	
FFNN2	0.953	0.975	0.639	0.489	0.936	0.967	0.810	0.589	
FFNN1	0.935	0.966	0.744	0.572	0.927	0.962	0.864	0.652	
GPR3	0.964	0.982	0.558	0.428	0.948	0.973	0.730	0.507	
GPR2	0.952	0.975	0.643	0.492	0.937	0.964	0.813	0.577	
GPR1	0.935	0.966	0.746	0.573	0.926	0.957	0.881	0.651	
DT3	0.980	0.989	0.427	0.322	0.946	0.970	0.750	0.527	
DT2	0.968	0.983	0.526	0.400	0.936	0.964	0.819	0.587	
DT1	0.938	0.967	0.733	0.565	0.923	0.956	0.893	0.661	
air2stream3	0.961	0.980	0.578	0.447	0.949	0.971	0.736	0.532	
air2stream2	0.912	0.952	0.865	0.682	0.892	0.938	1.041	0.822	
air2stream1	0.903	0.947	0.902	0.706	0.891	0.938	1.044	0.812	

**Table 8:** Performances of different model versions in modelling water temperature (T_w_) for Dischmabach station.

Model version	Training (Calibration)				Validation			
	R	d	RMSE (°C)	MAE (°C)	R	d	RMSE (°C)	MAE (°C)	
FFNN3	0.990	0.995	0.398	0.313	0.987	0.994	0.457	0.366	
FFNN2	0.985	0.992	0.504	0.387	0.984	0.992	0.507	0.394	
FFNN1	0.951	0.974	0.894	0.677	0.950	0.973	0.896	0.691	
GPR3	0.989	0.995	0.418	0.328	0.987	0.993	0.462	0.363	
GPR2	0.985	0.992	0.503	0.386	0.984	0.992	0.511	0.398	
GPR1	0.950	0.974	0.900	0.685	0.951	0.974	0.887	0.678	
DT3	0.994	0.997	0.325	0.255	0.983	0.991	0.536	0.418	
DT2	0.991	0.995	0.387	0.293	0.983	0.991	0.527	0.408	
DT1	0.966	0.982	0.746	0.553	0.941	0.969	0.972	0.734	
air2stream3	0.976	0.988	0.628	0.502	0.976	0.987	0.642	0.526	
air2stream2	0.967	0.983	0.740	0.588	0.965	0.982	0.759	0.607	
air2stream1	0.946	0.972	0.936	0.744	0.945	0.972	0.941	0.768	

**Table 9:** Performances of different model versions in modelling water temperature (T _w_) for Cedar station.

Model version	Training (Calibration)				Validation			
	R	d	RMSE (°C)	MAE (°C)	R	d	RMSE (°C)	MAE (°C)	
FFNN3	0.985	0.992	0.618	0.488	0.983	0.991	0.768	0.593	
FFNN2	0.976	0.988	0.777	0.614	0.979	0.990	0.800	0.623	
FFNN1	0.945	0.971	1.168	0.905	0.952	0.975	1.211	0.933	
GPR3	0.987	0.994	0.570	0.446	0.985	0.990	0.747	0.592	
GPR2	0.976	0.988	0.778	0.614	0.980	0.987	0.867	0.669	
GPR1	0.945	0.971	1.169	0.906	0.952	0.971	1.248	0.978	
DT3	0.992	0.996	0.448	0.347	0.985	0.989	0.779	0.602	
DT2	0.983	0.991	0.657	0.516	0.977	0.986	0.901	0.693	
DT1	0.946	0.972	1.156	0.895	0.951	0.972	1.243	0.955	
air2stream 3	0.983	0.992	0.649	0.511	0.983	0.991	0.743	0.588	
air2stream 2	0.976	0.988	0.780	0.615	0.979	0.987	0.869	0.682	
air2stream 1	0.966	0.983	0.922	0.733	0.967	0.981	1.047	0.839	

**Table 10:** Performances of different model versions in modelling water temperature (T_w_) for Fanno station.

Model version	Training (Calibration)				Validation			
	R	d	RMSE (°C)	MAE (°C)	R	d	RMSE (°C)	2c MAE (°C)	
FFNN3	0.982	0.991	0.962	0.751	0.985	0.993	0.948	0.754	
FFNN2	0.979	0.989	1.037	0.813	0.985	0.993	0.964	0.768	
FFNN1	0.958	0.978	1.458	1.153	0.968	0.984	1.383	1.101	
GPR3	0.981	0.990	0.973	0.760	0.986	0.988	1.154	0.931	
GPR2	0.978	0.989	1.048	0.820	0.985	0.987	1.213	0.994	
GPR1	0.957	0.978	1.462	1.159	0.969	0.979	1.514	1.241	
DT3	0.989	0.994	0.755	0.580	0.983	0.986	1.225	0.985	
DT2	0.985	0.992	0.871	0.677	0.982	0.986	1.265	1.021	
DT1	0.958	0.978	1.446	1.143	0.968	0.979	1.520	1.242	
air2stream3	0.988	0.994	0.777	0.607	0.987	0.990	1.091	0.869	
air2stream2	0.991	0.996	0.677	0.522	0.991	0.992	0.961	0.794	
air2stream1	0.985	0.993	0.867	0.689	0.984	0.989	1.128	0.912	

**Table 11:** Performances of different model versions in modelling water temperature (T_w_) for Irondequoit station.

Model version	Training (Calibration)				Validation			
	R	d	RMSE (°C)	MAE (°C)	R	d	RMSE (°C)	MAE (°C)
FFNN3	0.989	0.994	1.078	0.840	0.985	0.992	1.242	0.933	
FFNN2	0.987	0.994	1.138	0.876	0.986	0.993	1.195	0.926	
FFNN1	0.961	0.980	1.983	1.480	0.966	0.982	1.867	1.387	
GPR3	0.989	0.994	1.055	0.817	0.984	0.992	1.313	1.004	
GPR2	0.988	0.994	1.128	0.868	0.986	0.992	1.260	1.008	
GPR1	0.960	0.979	1.998	1.488	0.967	0.981	1.926	1.430	
DT3	0.993	0.997	0.825	0.633	0.984	0.991	1.339	1.068	
DT2	0.992	0.996	0.931	0.704	0.985	0.992	1.294	1.039	
DT1	0.962	0.980	1.949	1.453	0.965	0.980	1.976	1.459	
air2stream3	0.994	0.997	0.799	0.618	0.993	0.995	0.990	0.764	
air2stream2	0.994	0.997	0.793	0.605	0.993	0.996	0.939	0.702	
air2stream1	0.989	0.994	1.071	0.832	0.989	0.994	1.160	0.890	

**Figure 6:**
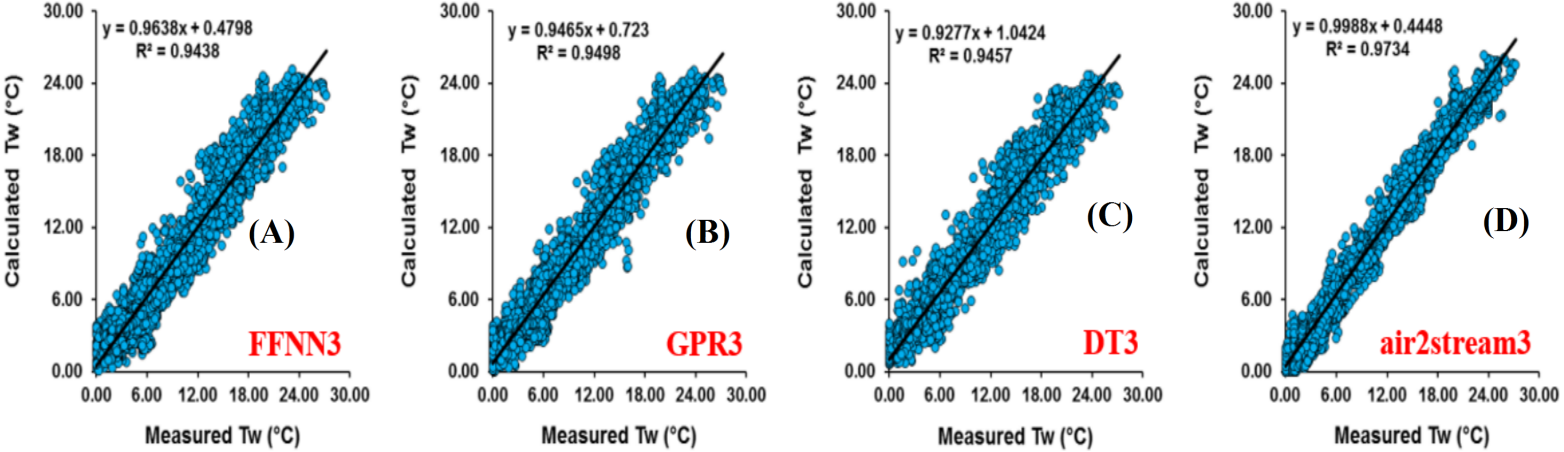
Scatterplot of measured versus calculated water *T_w_* at the Donji Miholjac River during the validation phase. (A) FFNN3. (B) GPR3. (C) DT3. (D) air2stream3.

Results at Donji Miholjac station are reported in Table 5. Figure 6 illustrates the scatterplot of measured and calculated values of *T*_*w*_ using the best models with *T*_*a*_, *Q*, and *DOY* as input variables. Two important points must be highlighted. Firstly, using the air2strteam, there is no significant difference between air2strteam1, air2strteam2 and air2strteam3. The R and d values were slightly improved between air2strteam1 and air2strteam3 (0.3% and 0.1%). In addition, the RMSE and MAE did not change significantly between the two models: 8.9% reduction for the RMSE, and 9.27% reduction for the MAE. Secondly, by comparing the accuracy of the three machine learning models, it is clear that the FFNN, GPR and DT models worked with slight difference. In addition, DT1 possess the lowest accuracy with high RMSE (2.732 °C) and MAE (2.134 °C), slightly higher than GPR1 (RMSE = 2.671 °C, MAE = 2.090 °C), while the FFNN1 was the best model with lowest RMSE and MAE values.

**Figure 7:**
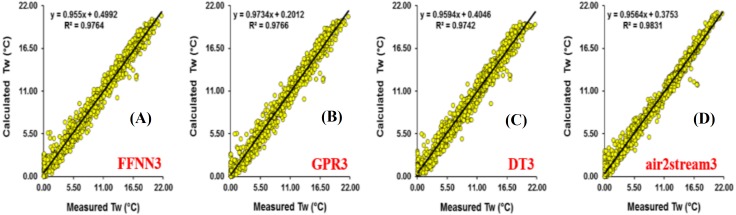
Scatterplot of measured versus calculated water *T_w_* at the Mentue River during the validation phase. (A) FFNN3. (B) GPR3. (C) DT3. (D) air2stream3.

The results at Mentue station are summarized in Table 6. Figure 7 illustrates the scatterplot of measured and calculated values of *T*_*w*_ using the best models with *T*_*a*_, *Q*, and *DOY* as input variables. As can be seen from Table 6, during the validation phase, the FFNN1, GPR1 and DT1 were practically identical. The difference in the RMSE and MAE values were small and marginal. The FFNN1 model was slightly more accurate than the GPR1 and DT1, with MAE of 0.982 °C and RMSE of 1.307 °C. However, using only *T*_*a*_ as input variable, air2strteam1 was more accurate than the three machine learning models. The difference in the performance of these algorithms for the prediction of *T*_*w*_ is discussed hereafter. The RMSE and MAE values were decreased from 1.307 °C to 0.933 °C (28.62%), and from 0.982 °C to 0.679 °C (30.86%) compared to FFNN1. Compared to the GPR1, the RMSE and MAE values were decreased from 1.311 °C to 0.933 °C (28.83%), and from 0.992 °C to 0.679 °C (31.55%), respectively. Finally, air2strteam1 decreased the RMSE and the MAE of the DT1 by 31.60% and 34.59%, respectively.

**Figure 8:**
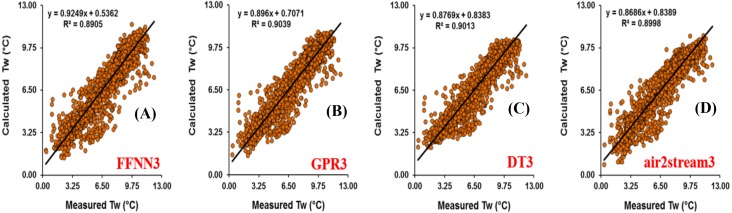
Scatterplot of measured versus calculated water *T_w_* at the Rhône River during the validation phase. (A) FFNN3. (B) GPR3. (C) DT3. (D) air2stream3.

Analysis of model performances at Rhône station (Table 7) showed that, for the models using only *T*_*a*_ as input variable, the three machine learning models were more accurate than the air2strteam model and FFNN1 (*R* = 0.927 and *d* = 0.962) and GPR1 (*R* = 0.926 and *d* = 0.957) performed the best and slightly better than the DT1 model (*R* = 0.923 and *d* = 0.956). According to the RMSE and MAE values, the air2strteam1 was the worst model with RMSE and MAE values equal to 1.044 °C and 0.812 °C, respectively. The FFNN1 gave the lowest values (RMSE = 1.044 °C and MAE = 0.812 °C) and improved the air2strteam1 by a 17.24% and 19.70% reduction in the RMSE and MAE values, respectively. Figure. 8 illustrates the scatterplot of measured and calculated values of *T*_*w*_ using the best models (version 3) at Rhône station.

**Figure 9:**
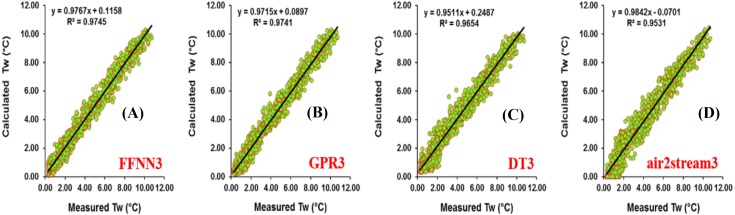
Scatterplot of measured versus calculated water *T_w_* at the Dischmabach River during the validation phase.

A summary of the model performances at Dischmabach station is provided in Table 8 and Fig. 9. It can be observed that GPR1 slightly outperformed FFNN1 and DT1 according to all measures. A RMSE of 0.887 °C was observed in estimated *T*_*w*_ using GPR1, while the respective values for FFNN1 and DT1 were 0.896 °C and 0.972 °C, respectively. GPR1 produced the highest R and d between the measured and calculated *T*_*w*_ (*R* = 0.951 and *d* = 0.974), while the air2strteam1 yielded R of 0.945 and d of 0.972, less than the values obtained using the GRP1. To show the importance of including the *DOY* and *Q* as inputs to the models, the corresponding performances are illustrated and compared. Comparing the FFNN2, GPR2 and DT2 models with the *DOY* as input in addition to the *T*_*a*_, it can be concluded that: (i) the three machine learning models provided the same accuracy with only marginal difference and (ii) the air2strteam2 yielded less accuracy compared to the FFNN2, GPR2 and DT2 models, with high RMSE and MAE, and low R and d values.

**Figure 10:**
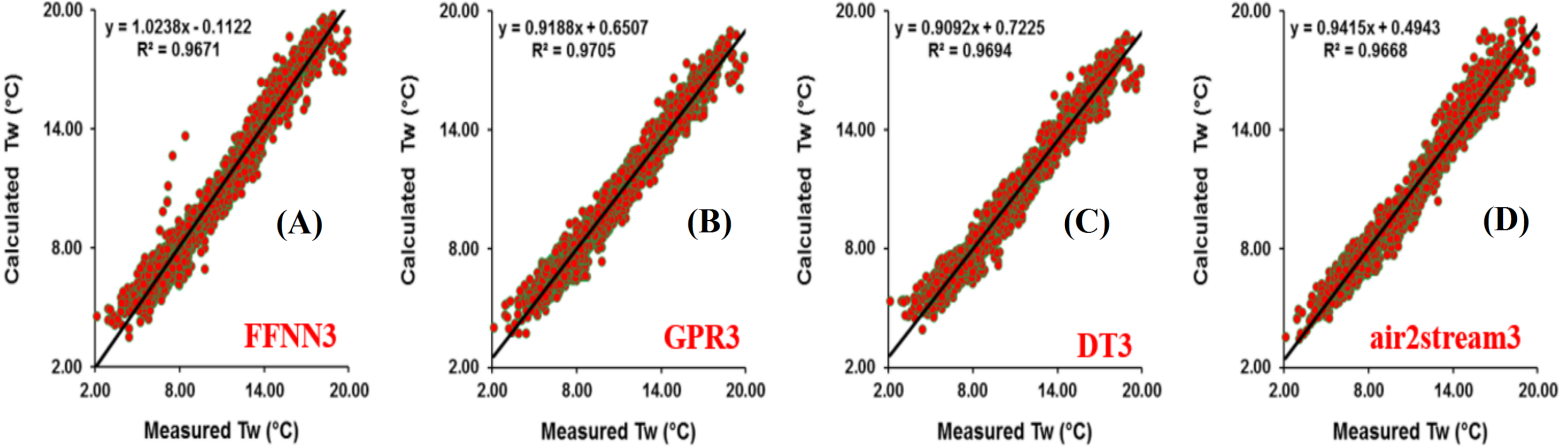
Scatterplot of measured versus calculated water *T_w_* at the Cedar River during the validation phase. (A) FFNN3. (B) GPR3. (C) DT3. (D) air2stream3.

Results obtained using the applied models at Cedar station are illustrated in Table 9. Figure 10 illustrates the scatterplot of measured and calculated values of *T*_*w*_ using the best models. Overall, using only the *T*_*a*_ as input, the maximum R and d values of 0.967 and 0.981 respectively during the validation phase were obtained using the air2strteam1 model. In addition, the RMSE and MAE values of the FFNN1, GRP1 and DT1 models obtained were 1.211 °C and 0.933 °C, 1.248 °C and 0.978 °C, and 1.243 °C and 0.955 °C, which were respectively 13.54% and 10.075%, 16.10% and 14.21%, and 15.76% and 12.14%, greater than the values obtained using the air2strteam1 model. Thus, it was demonstrated that the air2strteam1 model was more accurate than the machine learning models. With the inclusion of the *DOY* as input variable combined with the *T*_*a*_, the model performances varied accordingly and were significantly improved. The best accuracy was obtained with the FFNN2 model that had a significant decrease of the RMSE and MAE compared to the FFNN1 by 33.94% and 33.23%, respectively. Comparing the overall accuracy of the models using the version 2, it is clear from Table 9, that the four models provided the same accuracy with very marginal difference. Finally, as shown in Table 9, R and d values for the FFNN3, GPR3, DT3 and air2strteam3 reached up to 0.983, 0.985, 0.989, and 0.983, respectively, which were relatively higher compared to the values obtained using the versions 1 and 2.

**Figure 11:**
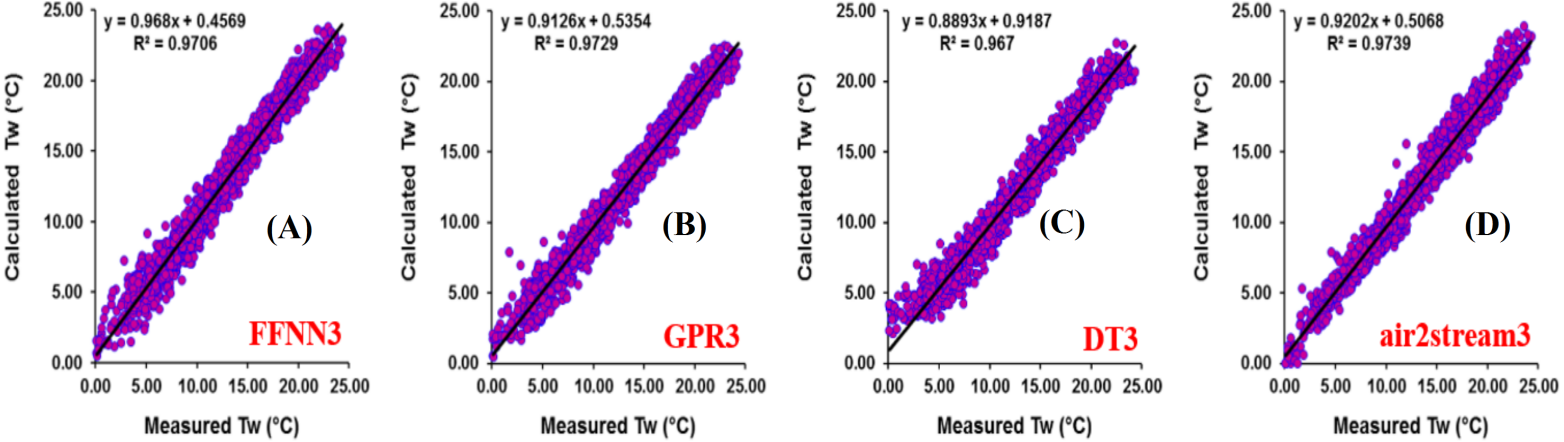
Scatterplot of measured versus calculated water *T_w_* at the Fanno River during the validation phase. (A) FFNN3. (B) GPR3. (C) DT3. (D) air2stream3.

Results at the Fanno station are reported at Fig. 11 and Table 10. The RMSE and MAE of the four models for this station were relatively low. It was indicated that the *T*_*w*_ calculated by air2strteam1 agreed well with the measured value, with R and d values of 0.984 and 0.989 during the validation phase. Compared to the machine learning models, air2strteam1 was the best model. Moreover, from the modeling accuracy reported in Table 10, the FFNN1, GPR1 and DT1 were less accurate than the air2strteam1. Analogously for the version 2 of models, air2strteam2 was the best model and yielded the highest R and d values (0.991 and 0.992), the lowest RMSE (0.961 °C) and MAE (0.794 °C), among the four developed models. On the other hand, except for the R values, the performances of the air2strteam2 was quite similar to FFNN2, with R, d, RMSE and MAE values being 0.985, 0.993, 0.964 °C and 0.768 °C, respectively. The DT2 model yielded the poorest accuracy with lowest R (0.982) and d (0.986) and highest RMSE (1.265 °C) and MAE (1.021 °C) values.

**Figure 12:**
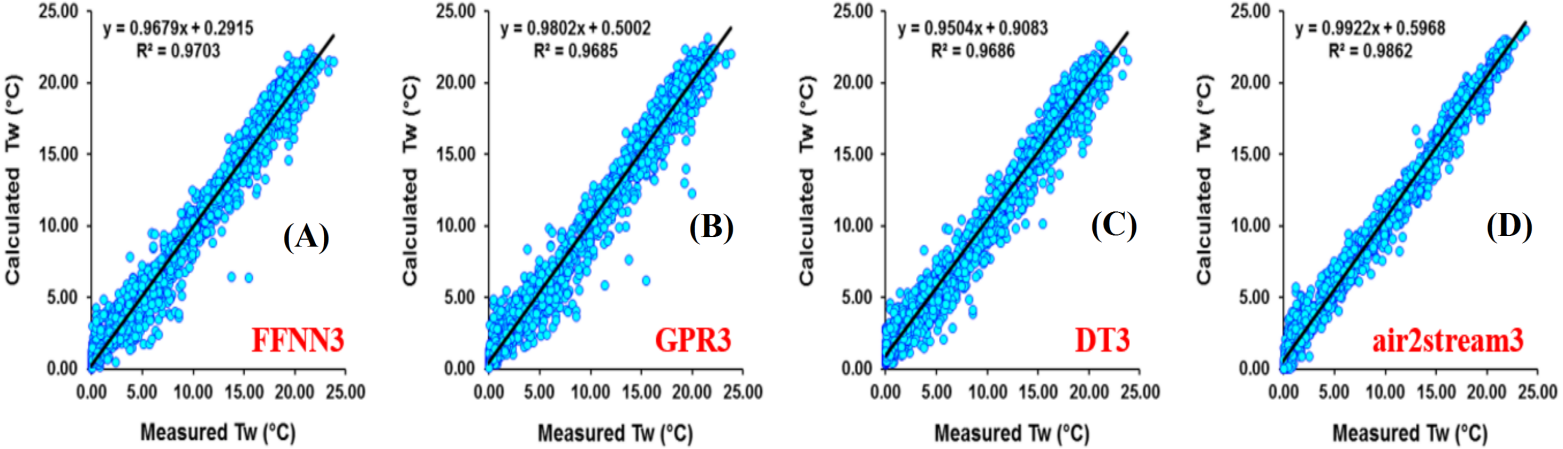
Scatterplot of measured versus calculated water *T_w_* at the Irondequoit River during the validation phase. (A) FFNN3. (B) GPR3. (C) DT3. (D) air2stream3.

Table 11 presents the performance metrics for the three machine learning models and air2strteam for Irondequoit River. Figure 12 illustrates the scatterplot of measured and calculated values of *T*_*w*_ using the best models. Superiority of the air2strteam for predicting *T*_*w*_ is evident for all three versions of models. During the validation phase, the superiority of the air2strteam1 is indicated by higher R (0.984) versus 0.966, 0.967, and 0.965 for FFNN1, GPR1 and DT1, respectively. This superior performance is confirmed by lower RMSE and MAE values (1.160 versus 1.867 °C, 1.926 °C, and 1.976 °C), and (0.890 versus 1.459 °C, 1.430 °C, and 1.387 °C) for FFNN1, GPR1 and DT1, respectively. As is shown in Table 11, air2strteam2 performs best, as indicated by higher R and d, and lower RMSE and MAE values. It is important to highlight the significant improvement of the models by the inclusion of the *DOY* as input variable. By adding the *DOY* to the model inputs, the R value was increased from 0.989 to 0.993 for air2strteam2, from 0.965 to 0.985 for DT1, from 0.967 to 0.986 for GPR2, and 0.966 to 0.986 for FFNN2, respectively (Table 11). The RMSE was decreased by 35.99%, 34.58%, 34.51% and 19.05%, when using FFNN2, GPR2, DT2 and air2strteam2, respectively. Similarly, the MAE was decreased by 33.23%, 29.51%, 28.78% and 21.12%, when using FFNN2, GPR2, DT2 and air2strteam2, respectively.

## Discussion

For the eight studied stations, the three machine learning models (FFNN1, GPR1 and DT1) performed comparatively when only *T*_*a*_ was used as predictor. R and d values were larger than 0.9, indicating good modelling performances. RMSE and MAE ranged from 0.733 to 2.574 °C and 0.553 to 2.015 °C in the calibration phase, and varied from 0.864 to 2.732 °C and 0.651 to 2.314 °C in the validation period. The air2stream1 model significantly outperformed the three machine learning models for most of the studied stations, except for Rhône at Sion and Dischmabach at Davos. Flow discharge (releases of cold water from high altitude hydropower reservoirs and snow melting from the high altitude areas) significantly impacted water temperature dynamics for Rhône River at Sion (Fig. 4D) and Dischmabach River at Davos (Fig. 4E). However, for air2stream1 model with three parameters (Eq. (4)), the effect of flow discharge was neglected (Toffolon & Piccolroaz, 2015; Piccolroaz et al., 2016), resulting in poor modelling performances.

When *DOY* was included as model input, modelling performances of the three machine learning models (FFNN2, GPR2 and DT2) dramatically improved in term of higher R, d and lower RMSE and MAE values (Tables 4–11), which confirmed the results of Heddam (2016), Heddam & Kisi (2017), and Zhu et al. (2019). In their researches, the inclusion of the components of the Gregorian calendar significantly improved the performance of the machine learning models in the case of dissolved oxygen and water temperature modelling. R and d values varied from 0.952 to 0.993 and 0.975 to 0.996 in the calibration phase, while for the validation phase, R and d values ranged from 0.936 to 0.986 and 0.964 to 0.993 respectively. RMSE and MAE ranged from 0.387 to 1.481 °C and 0.293 to 1.178 °C in the calibration phase, and varied from 0.507 to 1.783 °C and 0.394 to 1.422 °C in the validation phase. Modelling results indicate that the inclusion of *DOY* is significant for river water temperature simulation since it provides additional information on the seasonality of the river thermal dynamics compared to that encapsulated in the *T*_*a*_ time series. The air2stream2 model performed better than the air2stream1 model, which indicates that the sinusoidal annual term in Eq. (2) is important for river water temperature prediction since this annual periodicity can mimic the effect of lateral inflows and heat fluxes (Toffolon & Piccolroaz, 2015; Piccolroaz et al., 2016). The air2stream2 model outperformed the three machine learning models in most of the studied stations, except for Rhône at Sion, Dischmabach at Davos and Cedar River, which were impacted by cold water releases from high altitude hydropower reservoirs or snow melting.

The comparison between the improvement in river *T*_*w*_ prediction obtained using model version 3 and version 2 indicated that the inclusion of flow discharge provided a lower gain than including *DOY* for all the cases (Tables 4–11). This result indicates that in the proposed models *Q* plays a minor role, and the addition of *DOY* significantly contributes to better capture the seasonal pattern of river thermal dynamics. The relative importance of *Q* increases for the studied station in the Rhône, Dischmabach and Cedar rivers. The results also suggested that the role of *Q* on *T*_*w*_ modelling is more relevant for high altitude rivers impacted by cold water release from hydropower reservoirs or snow melting. However, for lowland hydrological stations impacted by hydropower reservoirs, such as the Drava River at Botovo, the influence of *Q* is not that remarkable. The air2stream3 model outperformed the three machine learning models in most of the studied stations, except for Rhône at Sion, Dischmabach at Davos and Cedar River.

For all the three model versions, DT model outperformed FFNN and GPR models in the calibration phase, while for the validation phase, its performance slightly decreased. Generally, FFNN model performed slightly better than GPR model, while the overall difference can be neglected. The RMSE values for the eight studied stations range from 0.956 to 1.366 °C, 1.014 to 1.69 °C, 0.611 to 0.947 °C, 0.427 to 0.763 °C, 0.325 to 0.536 °C, 0.448 to 0.779 °C, 0.755 to 1.225 °C, and 0.825 to 1.339 °C respectively for all the version 3 machine learning models, which are reasonable compared with Jackson et al. (2018) (1.57 °C) and Sohrabi et al. (2017) (1.25 °C), and far better than that of Temizyurek and Dadaser-Celik (2018) (2.10–2.64 °C). Overall, the three machine learning models (FFNN3, GPR3 and DT3) performed well for river water temperature modelling.

## Conclusions

In this study, different versions of FFNN, GPR and DT models were developed to simulate daily river water temperatures using *T*_*a*_, *Q*, and *DOY* as predictors. The models were assessed in various river systems, and modelling results were compared with air2stream model. For the eight studied river stations, the FFNN, GPR and DT models performed similarly when only *T*_*a*_ was used as predictor. When *DOY* was included as input, modelling performances of the FFNN, GPR and DT models dramatically improved in term of higher R, d and lower RMSE and MAE values. The inclusion of *Q* provided a lower gain than including *DOY* for all the cases, which indicates that in the proposed models *Q* plays a minor role. However, the relative importance of *Q* increases for the studied station in the Rhône River, Dischmabach River and Cedar River, which suggested that the role of flow discharge on river water temperature modelling is more relevant for high altitude rivers impacted by cold water releases from hydropower or snow melting. The air2stream model outperformed the three machine learning models in most of the studied rivers except for the Rhône River, Dischmabach River and Cedar River. For the eight studied river stations, RMSE values of MLPNN3, GPR3 and DT3 models ranged from 0.398 to 1.690 °C, 0.418 to 1.592 °C, and 0.325 to 1.668 °C respectively. For the Mentue River, Rhône River, Dischmabach River, and Cedar River, the RMSE values are lower than 1.0 °C. Overall, FFNN, GPR and DT models performed well for river water temperature modelling.

##  Supplemental Information

10.7717/peerj.7065/supp-1 Dataset S1 The observed data used in this study including daily air temperature, water temperature and river discharge

10.7717/peerj.7065/supp-2 Code S1 The code used for ANN models in the Matlab platform

## Additional Information and Declarations

### Data Availability

The following information was supplied regarding data availability: Raw data compiled from the USGS website (https://waterdata.usgs.gov/nwis/inventory) are available as Dataset S1. The data we used in this study can also be found at the USGS website: https://waterdata.usgs.gov/nwis/inventory. Readers can input the USGS station number (for example, for Cedar River, users can input 12119000) and select the data for output. In this study, we selected daily water temperature and flow discharge. The data can be output to a TXT file for use.
